# Serum lipid profile in relation to free thyroxine and the effect of levothyroxine treatment on lipids in patients with isolated hypothyroxinemia during pregnancy: a single-center retrospective study

**DOI:** 10.1186/s12944-022-01744-5

**Published:** 2022-12-19

**Authors:** Yunyi Xu, Yiqi Zhao, Xiaoqin Xu, Qiqi Yan, Liwei Yang

**Affiliations:** 1 Department of Obstetrics and Gynecology, The Second School of Clinical Medicine, Zhejiang Chinese Medical University, Hangzhou, Zhejiang China; 2 Center for Reproductive Medicine, Department of Obstetrics, Zhejiang Provincial People’s Hospital (Affiliated People’s Hospital, Hangzhou Medical College), Hangzhou, Zhejiang China; 3 Department of Gynecology & Obstetrics, Hangzhou Fuyang Women and Children Hospital, Hangzhou, Zhejiang China

**Keywords:** Pregnancy, Isolated hypothyroidism, Free thyroxine, Serum lipid profile, Levothyroxine treatment, Body mass index

## Abstract

**Background:**

Thyroid function is widely considered a lipid metabolism regulator. However, studies on lipid metabolism in pregnant women with low free thyroxine (FT_4_) levels are limited and inconclusive. Furthermore, the association between maternal FT_4_ deficiency and adverse lipid metabolic parameters is unknown. Therefore, we aimed to investigate this association and the effects of levothyroxine (L-T_4_) treatment on these metabolic indicators.

**Methods:**

This retrospective study included 164 patients with isolated hypothyroidism (IH) (FT_4_ levels below the 5^th^ percentile with normal thyroid stimulating hormone levels according to the gestational-specific reference range) and 407 euthyroidism patients (control group who had regular antenatal examinations at Zhejiang Provincial People's Hospital, Hangzhou, China) between January 1, 2019, and December 31, 2020. Patients with IH were divided into levothyroxine (L-treatment group, *n* = 77) and dietary iodine supplement treatment groups (dietary treatment group, *n*=87) according to the hospital’s treatment policy and clinical experience. The intervention lasted for at least 8 weeks. Metabolic indicators, including thyroid function and lipid parameters, were collected at least twice before and after the intervention. Other data collected included maternal age, history of abortion, prepregnancy BMI, and gestational weight gain (Fig. 1).

**Results:**

Compared with the control group, Patients with IH had a higher degree of dyslipidemia, reflected in elevated total cholesterol (TC), triglycerides (TG), low-density lipoprotein cholesterol (LDL-C), and apolipoprotein B (Apo B) levels. In IH patients, an inverse correlation was found between FT_4_ and TG levels, which remained after adjusting for prepregnancy BMI. The L-treatment group demonstrated a significantly slower rate of hypercholesterolemia progression during pregnancy than the dietary treatment group. In addition, there was a relationship between the therapeutic effect and the degree of disease, with the main factors being FT_4_, TSH and TG levels prior to starting treatment.

**Conclusions:**

Low FT_4_ levels were associated with elevated blood lipid levels. Serum FT_4_ and lipid levels in patients could be improved by medical intervention.

## Background

Thyroid hormones play an important role in regulating dynamic energy balance [1]. Hypothyroidism is a common endocrine problem during pregnancy due to alterations in various hormones. In such patients, an increased thyroid stimulating hormone (TSH) level is not equivalent to decreased free thyroxine (FT_4_) concentrations. With relatively low FT_4_ concentrations, ignoring immunological factors, isolated hypothyroidism (IH) [2] is associated with only mild clinical symptoms and aberrant laboratory parameters. Its incidence among pregnant women ranges from 1.3% to 23.9% [3–5]. Numerous studies have documented negative neurocognitive outcomes in neonates, including lower intelligence quotient and delayed language and motor function development [6–11]. Additionally, it may affect birth weight [12, 13] and cause premature delivery [14]; however, these conclusions remain controversial. Recently, patients with IH have received increasing attention, and experts have begun to explore their metabolic levels. Unfavorable metabolic parameters may explain adverse pregnancy outcomes to a certain extent [4, 15]. Timely and targeted treatment is necessary to prevent the negative effects of hormonal changes in pregnant women and fetuses.

Furthermore, pregnancy-related hormonal changes can affect the synthesis and metabolism of lipids in the liver, resulting in the physiological elevation of serum lipid profiles. Consequently, these elevated levels double the risk of gestational diabetes mellitus [16], hypertension, intrahepatic cholestasis [17], and preterm birth. [18]. Maternal blood lipid levels and the birth weight of neonates are closely associated [19, 20]. Furthermore, congenital cardiac disease in children is associated with hyperlipidemia during the first trimester [21]. Therefore, doctors have strengthened the management of blood lipid levels and weight in pregnant women to decrease the likelihood of unfavorable outcomes.

Dyslipidemia has gradually become a common feature of thyroid dysfunction. However, studies on lipid metabolism in the general IH population are few and inconclusive and are even rarer in pregnant women. When thyroid hormones in pregnant women fluctuate within the normal range, lipid metabolism is in a dynamic balance, ensuring that the body's requirement for fat is met while avoiding the accumulation of excess fat that can cause other pathological conditions. Research on lipid metabolism in patients with IH is limited. Mehran pointed out that FT_4_ is closely related to metabolic indicators, such as TC and TG levels, and lower FT_4_ levels may increase the risk of developing metabolic syndrome [22]. During pregnancy, lipid profile alterations are complex. Knight noted that FT_4_ levels were distinctly negatively associated with BMI and TG but not with TC; however, TSH levels were not correlated with any of these metabolic parameters [4]. Hong concluded that the levels of TC, TG, HDL, and LDL in pregnant women with IH were higher than those in the normal group [23]. In a previous study conducted in our unit, TG levels were elevated in IH in the second and third trimesters compared with normal controls [24]. However, a retrospective investigation conducted in China did not identify dyslipidemia in patients with IH. Since it is not known whether maternal FT_4_ deficiency during pregnancy is associated with adverse lipid metabolic parameters, our study aimed to investigate this association.

Levothyroxine (L-T_4_) is currently recognized as the most effective and convenient drug for treating hypothyroidism. The 2017 American Thyroid Association (ATA) guidelines [2] emphasized the lack of evidence, including linking hypothyroxinemia (HT) in pregnancy to fetal development and the benefits of interventions to treat HT [25]. Furthermore, L-T_4_ treatment can result in overtreatment and overdose, which are harmful. Hence, multinational guidelines do not routinely recommend the administration of L-T_4_ to patients with IH. Additionally, these guidelines highlighted the inferiority of the recommended evidence and concluded that L-T_4_ effectiveness needs further evaluation. However, studies have shown that L-T_4_ supplementation reverses lipid changes induced by hypothyroidism, especially in patients with TSH levels > 10 mIU/L [26]. However, no study has evaluated the effect of L-T_4_ on lipid metabolism in pregnant women with IH worldwide. Additionally, hyperlipidemia during pregnancy has no effective or safe drugs [27]. Therefore, we aim to explore the comprehensive effect of L-T_4_ on serum lipid profile in pregnant patients with IH, which will guide the development of clinical work.

## Materials and methods

### Study design and participants

This was a single-center retrospective study. We collected information on pregnant women who received perinatal care at Zhejiang Provincial People's Hospital (Hangzhou, China) between January 1, 2019, and December 31, 2020, (*n* = 6710). In 2017, the ATA guidelines for diagnosing and managing thyroid disease during and after pregnancy defined HT as subnormal serum FT_4_ levels (approximately the 5^th^ percentile) and normal serum TSH levels (2.5^th^ **–** 97.5^th^ percentiles). In this case, IH is the proportion of patients with HT who are negative for thyroglobulin and thyroid peroxidase antibodies.

We used data from the second and third trimesters for this study because the current policy in China encourages pregnant women to undergo early prenatal check-ups (before 24 weeks of gestation) in community health service institutions, and thyroid function fluctuates greatly in the first trimester of pregnancy. According to the hospital’s different established reference ranges of thyroid function in the second and third trimesters, we included 164 patients with gestational IH who met the following criteria: (1) single pregnancy, (2) 18**–**40 years old, (3) no prepregnancy thyroid disease, and no prior history of anti-thyroid drugs intake, radioactive ^131^ I therapy, and thyroid surgery, (4) no history of significant organ disease or malignancy, and (5) no chronic disease including hypertension, diabetes, autoimmune disease, mental illness, or other major diseases such as cardiovascular diseases.

**Fig. 1:**
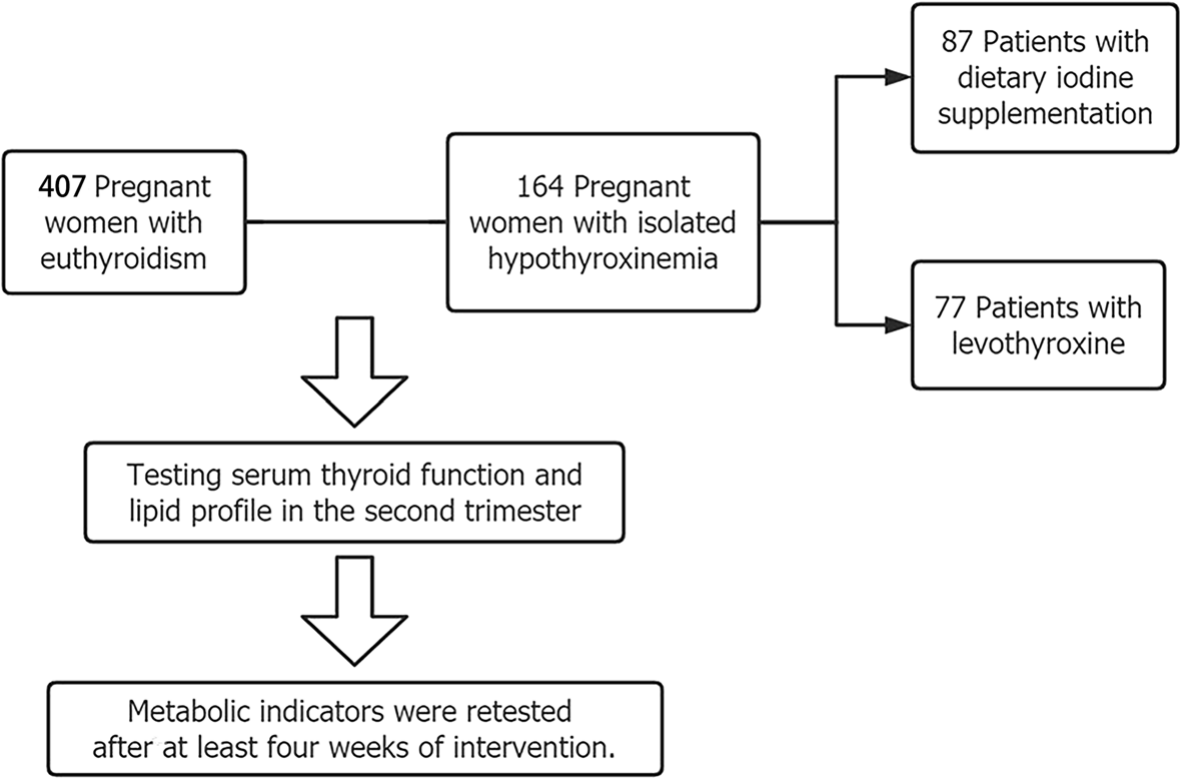
Flow chart of this study

All patients with IH were advised to receive more dietary iodine supplementation, such as kelp. Owing to the hospital’s treatment policy and clinical experience, patients voluntarily accepted or refused drug treatment. Patients who refused drugs, due to difficulty maintaining medication or fear of side effects, constituted the dietary iodine supplement group (dietary treatment group, *n* = 87). Patients treated with L-T_4_ were classified into the levothyroxine treatment group (L-treatment group, *n* = 77). The initial dosage primarily depended on serum FT_4_ levels. Clinicians recommended an initial dose of 25 μg L-T_4_ and adjusted the maintenance dosage according to the results retested every 4 weeks. Furthermore, we randomly selected controls (*n* = 407) who were pregnant women with normal range thyroid function (euthyroidism). Each case was matched to the three controls in terms of age, gravidity, and parity. It was recommended to recheck all individual cases for metabolic indexes, including thyroid function and lipid indicators, at least 8 weeks later (Fig. 1). The ethics committee approved the study of Zhejiang Provincial People's Hospital (Number: QT2022364).

### Data collection

Metabolic parameters, including serum thyroid function and lipid profile of patients with IH and euthyroidism during pregnancy, were collected at least twice before and after the intervention. Blood lipid profiles included total cholesterol (TC), triglyceride (TG), low-density lipoprotein cholesterol (LDL-C), high-density lipoprotein cholesterol (HDL-C), apolipoprotein A1 (Apo A1), and apolipoprotein B (Apo B). We also collected data on several general characteristics, such as maternal age, history of abortion, progestational BMI, and gestational weight gain.

### Statistical analysis

All statistical analyses were performed using IBM SPSS Statistics version 25 for Windows (Stata Corp., College Station, TX, USA). Data were expressed as the mean ± standard deviation or median (interquartile range). Data related to thyroid function were not normally distributed. After transforming the data to approximate a normal distribution, a *t* test was used to compare the differences between the groups. Lipid-related indicators were mostly normally distributed, and a *t* test was used to investigate alterations in blood lipid profiles at low thyroxine levels and changes before and after treatment. The levothyroxine intervention effects on thyroid function and lipid profiles in IH patients were also assessed by multiple linear regression models after adjusting for potential confounders.

## Results

We observed no significant differences in maternal age, gravidity, or parity between the case and control groups. Compared with the controls, patients with IH had significantly higher levels of TC (6.28 *vs* 6.02 mmol/L, *P* = 0.015), TG (2.44 *vs* 2.01 mmol/L, *P* < 0.001), LDL-C (3.18 *vs* 3.02 mmol/L, *P* = 0.047) and Apo B (1.06 *vs* 1.00 mmol/L, *P* = 0.002) (Table 1).

**Table 1:** General characteristics of pregnant women with IH and controls

**Clinical characteristics** **Laboratory indicators**	**IH Group,** **(*n* = 164)**	**Control Group,** **(*n* = 407)**	***P* Value**
**TSH**,average,mIU/L	1.96±0.67	1.88±0.72	0.079
**FT**_**4**_,average,ng/L	4.88±0.25	6.73±0.75	<0.001
**TC**,average,mmol/L	6.28±1.21^b^	6.02±0.86	0.015
**TG**,median,mmol/L	2.44(2.06,3.09)^b^	2.01(1.63,2.50)	<0.001
**HDL-C**,average,mmol/L	1.85±0.40	1.90±0.36	0.282
**LDL-C**,median,mmol/L	3.18±0.93^b^	3.02±0.71	0.047
**Apo A1**,average,mmol/L	2.16±0.34	2.15±0.27	0.721
**Apo B**,average,mmol/L	1.06±0.22^b^	1.00±0.20	0.002
**Maternal age**,median, years	28.50(26.00,31.00)	28.00(25.00,31.00)	0.500
**Gravidity**,median	2.00	2.00	0.379
**Parity**,median	0.00	0.00	0.873
**Primipara**,n(%)	107(65.20%)	266(65.40%)	0.980
**Prepregnancy BMI**^c^, median, kg/m^2^	21.99(20.12,23.86)	20.56(18.82,22.48)	<0.001
Underweight,n(%)	10(6.6%)	80(20.8%)	
Normal weight,n(%)	108(71.1%)	242(62.9%)	
Overweight,n(%)	29(19.9%)	53(13.8%)	
Obesity,n(%)	5(3.3%)	10(2.6%)	
**Gestational BMI gain** (**ΔBMI**^d^),median,kg/m^2^	5.65(4.69,7.42)	5.21(4.19,6.01)	<0.001
**Gestational weight gain** (**GWG**),median,kg	15.00(12.00,19.00)	13.00(11.00,15.00)	<0.001

^a^The diagnostic criteria for IH were that only FT_4_ was below the 5^th^ percentile with a normal TSH level (2.5th-97.5th). The reference ranges for this study center specifically were FT_4_ ≤ 5.23 ng/L and TSH 0.48-3.91 mIU/L in the second trimester; FT_4_ ≤ 5.40 ng/L and TSH 0.42-4.33 mIU/L in the third trimester during pregnancy. ^b^Compared with the control group, the IH group displayed higher levels of dyslipidemia, mainly reflected in elevated TC, TG, LDL-C and Apo B. ^c^Categories according to World Health Organization(WHO): underweight: < 18.5 kg/m^2^, normal: 18.5≤x<24 kg/m^2^, overweight: 24≤x<28 kg/m^2^, obesity≥28 kg/m^2^. ^d^ΔBMI, BMI at delivery minus prepregnancy BMI

Differences in prepregnancy BMI levels (21.99 *vs* 20.56 kg/m^2^, *P* < 0.001) were observed between patients with IH and euthyroidism. Gestational BMI gain (ΔBMI, 5.65 *vs* 5.21 kg/m^2^, *P* < 0.001) and gestational weight gain (GWG, 15.00 *vs* 13.00 kg, *P* < 0.001) were significantly higher in the IH group than in the control group. Additionally, when stratified by prepregnancy basal BMI levels (underweight: <18.5 kg/m^2^, normal: 18.5≤x<24 kg/m^2^, overweight: 24≤x<28 kg/m^2^, obesity: ≥28 kg/m^2^), FT_4_ (4.95, 4.89, 4.85, 4.79 ng/L) and HDL-C (1.90, 1.87, 1.88, 1.72 mmol/L) levels followed a decreasing trend with increasing BMI in patients with IH. However, no statistically significant differences were seen. Due to the high BMI levels in IH, the difference between FT_4_ and partial serum lipid levels was still statistically significant after adjusting for prepregnancy BMI levels (Table 2).

**Table 2:** Serum lipid profile of IH and controls after adjusting for prepregnancy BMI

**Laboratory indicators**	**Adjusted** **IH group,** **(*n* = 151)**	**Adjusted** **Control group,** **(*n* =151)**	***P* Value**
**ΔBMI**,median,kg/m^2^	5.65(4.69,7.44)	5.08(4.01,5.86)	<0.001
**GWG**,median,kg/m^2^	15.00(12.00,19.00)	13.00(10.00,15.00)	<0.001
**TSH**,average,mIU/L	1.95±0.68	1.87±0.71	0.356
**FT**_**4**_,average,ng/L	4.88±0.25	6.76±0.70	<0.001
**TC**,average,mmol/L	6.26±1.20	5.91±0.80	0.003
**TG**,median,mmol/L	2.44(2.06,3.10)	2.06(1.64,2.50)	<0.001
**HDL-C**,average,mmol/L	1.87±0.41	1.86±0.36	0.835
**LDL-C**,average,mmol/L	3.14±0.89	2.92±0.58	0.011
**Apo A1**,average,mmol/L	2.18±0.34	2.11±0.27	0.061
**Apo B**,average,mmol/L	1.05±0.22	0.98±0.17	0.001

**Fig. 2:**
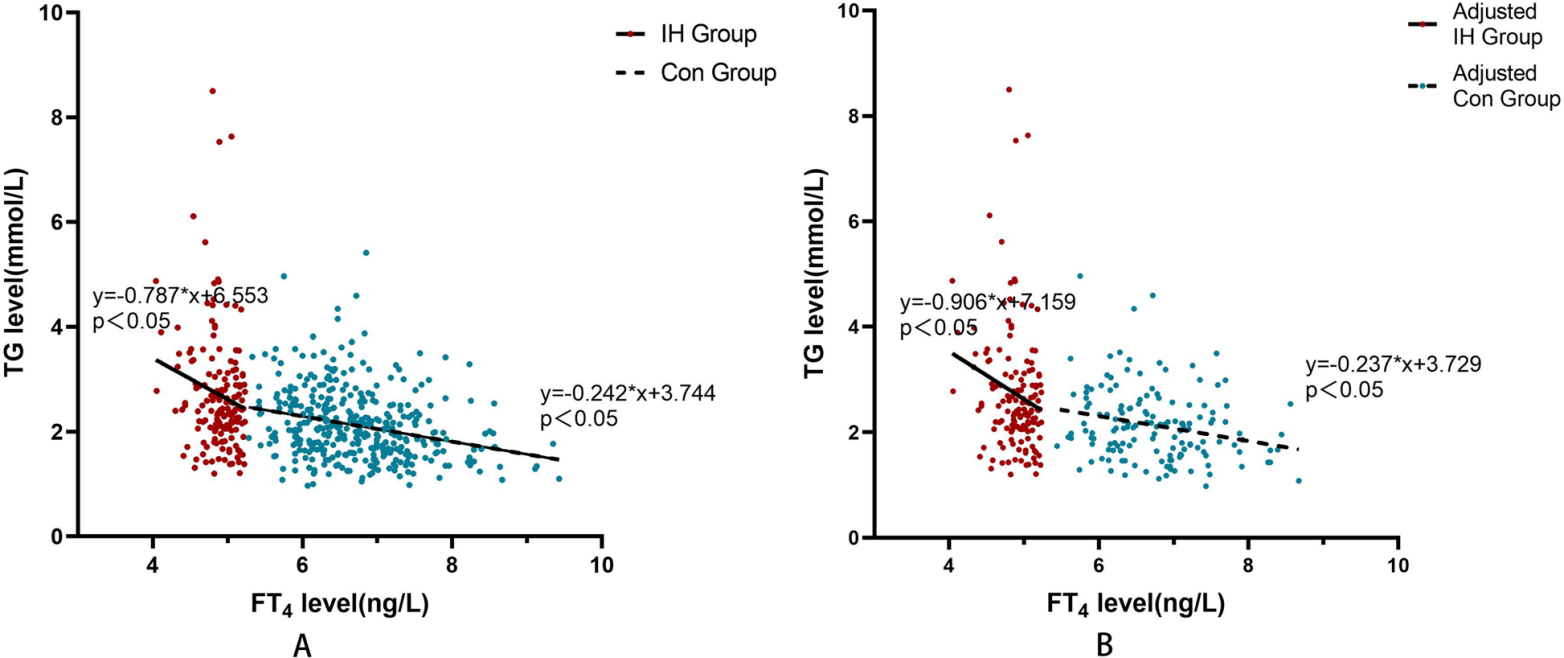
Correlation between FT_4_ and TG levels in IH and control groups before and after adjusting prepregnancy BMI. **A** In IH patients, the linear regression model showed that serum TG levels were related to FT_4_ levels (y= -0.787x + 6.553, *P* = 0.030, R^2^ = 0.029). In controls, the model was also established (y= -0.242x + 3.744, *P <* 0.001, R^2^ = 0.073). **B** After adjusting for prepregnancy BMI, the linear regression model was still found that TG levels were related to FT_4_ levels (y= -0.906x + 7.159, *P* = 0.018, R^2^ = 0.03). Similarly, in the control group this association was found (y= -0.237x + 3.729, *P* = 0.003, R^2^ = 0.06)

A negative correlation was found between FT_4_ and TG levels (*r* = -0.158, *P* = 0.043) in patients with IH and euthyroidism (*r* = -0.283, *P* < 0.001). Additionally, serum TG levels were significantly correlated with FT_4_ levels. These correlations remained significant after adjusting for prepregnancy BMI (Fig. 2).

**Fig. 3:**
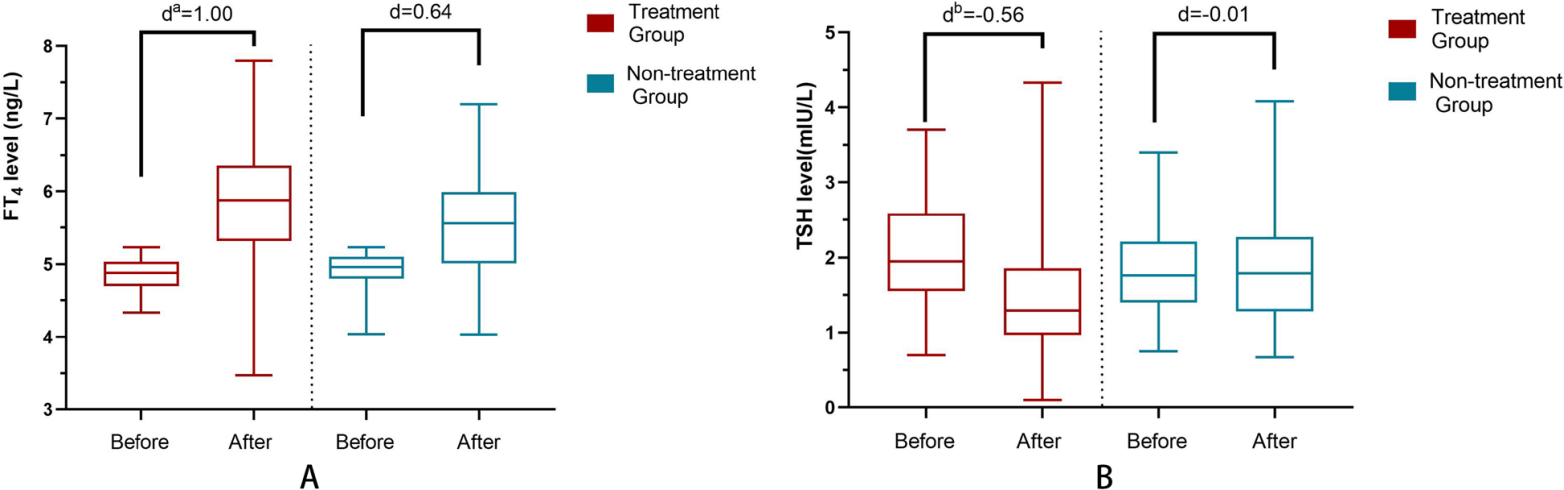
Alterations of FT_4_ and TSH levels before and after different interventions in IH patients. **A**^a^ The indicators in the L-T_4_ group (d = 1.00 ng/L, average) were more significant than thoes in the nontreatment group (d = 0.64 ng/L) (*P* = 0.003). **B**^b^ The alterations in TSH levels in the L-T_4_ group (d= -0.56 mIU/L, average) were more significant than those in the nontreatment group (d= -0.01 mIU/L) (*P*<0.001)

Table 3 shows the course of thyroid function in patients with IH during pregnancy for the two intervention. In the dietary treatment group (*n* = 87), although no medication was used, there was a significant increase in FT_4_ (4.90 to 5.54 ng/L, *P* < 0.001) but little alteration in TSH (1.86 to 1.86 mIU/L, *P* = 0.905). After levothyroxine intervention(*n*=77), the levels of FT_4_ (4.86 to 5.86 ng/L, *P*<0.001) and TSH (2.07 to 1.50 mIU/L, *P*<0.001) were significantly improved, and the degree of improvement was more significant than that in the dietary treatment group(FT_4:_ -0.56 *vs* -0.01 mIU/L, *P*<0.001, TSH_:_ 1.00 *vs* 0.64 ng/L, *P*=0.003). Furthermore, the rate of return to normal thyroid function was observably higher in the L-treatment group than in the dietary treatment group (*P* = 0.008) (Fig. 3).

**Table 3:** Thyroid function before and after treatment with L-T_4_ in patients with IH

**Thyroid function**	**L-treatment group,** **(*n*= 77)**		**Dietary treatment group,** **(*n* = 87)**	
	Before	After	Before	After
**TSH**,average,mIU/L	2.07±0.75^a^	1.50±0.80^b^	1.86±0.58	1.86±0.77
**FT**_**4**_,average,ng/L	4.86±0.22^b^	5.86±0.76^c^	4.90±0.26	5.54±0.70

^a^The basic serum TSH and FT_4_ levels of the two groups were similar (*P* = 0.060, *P* = 0.287). ^b^TSH levels in the L-treatment group were significantly different from the dietary treatment group data after the intervention (1.50 *vs* 1.86 mIU/L, *P* = 0.005). ^c^FT_4_ levels in the L-treatment group were statistically higher than those in the dietary treatment group data after the intervention (5.86 *vs* 5.54 ng/L, *P* = 0.005)

Moreover, the lipid profile showed interesting changes in response to the L-T_4_ intervention. Changes in serum lipid levels in the control group were used as the fundamental levels of raised blood lipids. These fundamental levels made comparing the differences in serum lipid levels in each case rigorous and scientific. Differences in TC (*P* = 0.003), TG (*P* = 0.016), LDL-C (*P* < 0.001), and Apo B levels (*P* = 0.003) after the intervention significantly differed among the three groups (Table 4). The increasing level of TC in the dietary-treated group was close to that in the control group; however, its degree in the treatment group was significantly lower (*P* = 0.035). The degree of alteration of TG, HDL-C and Apo A1 levels in the L-treatment and dietary treatment groups was greater than that in the control group, with no significant difference between the groups. The increasing rate of LDL-C levels in pregnancy after different intervention modalities was lower in patients with IH than that in controls.

**Table 4:** Lipid parameters of the subjects in the intervention study at baseline and at last

**Serum lipid profile**	**L-treatment group,** **(*n*= 77)**			**Dietary treatment group,** **(*n* = 87)**			**Con group,** **(*n* = 407)**		
	Before	After	***Δ***^**a**^	Before	After	***Δ***	Before	After	***Δ***^***b***^
**TC**^c^,average,mmol/L	6.41	6.45	***0.05***	6.16	6.42	***0.26***	6.02	6.33	***0.31***
**TG**,median, mmol/L	2.41	3.01	***0.72***	2.44	3.26	***0.68***	2.01	2.55	***0.49***
**HDL-C**,average,mmol/L	1.91	1.75	***-0.17***	1.80	1.65	***-0.15***	1.89	1.77	***-0.13***
**LDL-C**,average,mmol/L	3.24	3.34	***0.09***	3.13	3.19	***0.06***	3.02	3.35	***0.32***
**Apo A1**,average,mmol/L	2.23	2.20	***-0.07***	2.10	2.05	***-0.04***	2.14	2.13	***-0.01***
**Apo B**,average,mmol/L	1.07	1.17	***0.11***	1.06	1.12	***0.06***	1.00	1.13	***0.12***

^a^**Δ**: Postintervention lipid indexes minus preintervention indexes. ^b^The lipid indicators in the control group were used as the fundamental levels of raised blood lipids during pregnancy. ^c^Before intervention, the rise range of TC was much higher in the dietary-treatment group (0.26 mmol/L) than in the L-treatment group (0.05 mmol/L) after the intervention (*P* = 0.035)

Based on the specific diagnostic criteria for IH, we defined the 90^th^ percentile for FT_4_ levels as the cutoff, with the low FT_4_ level subgroup (FT_4_ < 4.707 ng/L, *n* = 20) having a greater rise in FT_4_ levels after L-T_4_ treatment (1.38 *vs* 0.87 ng/L, *P* = 0.011). Similarly, the high TSH level subgroup (TSH: 2.5 **–**3.91 mIU/L, *n* = 20) showed a greater improvement in TSH (-1.20 *vs* -0.34 mIU/L, *P* = 0.002) and FT_4_ levels (1.05 *vs* 0.98 ng/L, *P* = 0.718) after drug intervention. However, the degree of decline in HDL-C levels (-0.26 *vs* -0.13 mmol/L, *P* = 0.033) was more pronounced. Based on the range of dyslipidemia during pregnancy, the low TG level subgroup (TG < 2.41 mmol/L, *n* = 38) showed a significantly greater decrease in HDL-C (-0.23 *vs* -0.10 mmol/L, *P* = 0.017) after treatment with L-T_4_. However, in the high TG level subgroup (TG ≥ 2.41 mmol/L, *n* = 39), we found worsening thyroid function (TSH: -0.45 *vs* -0.68 mIU/L, *P* = 0.213, FT_4_: 0.98 *vs* 1.02 ng/L, *P* = 0.850) and worse lipid-related indicators, mainly in TC (0.10 *vs* -0.02 mmol/L, *P* = 0.406), TG (0.76 *vs* 0.69 mmol/L, *P* = 0.660), LDL-C (0.17 *vs* < 0.01 mmol/L, *P* = 0.191), and Apo B (0.12 *vs* 0.09 mmol/L, *P* = 0.508) levels, although there was no significant difference.

After excluding confounding factors such as parity, gravidity, maternal age, and prepregnancy BMI, we established that L-T_4_ intervention could significantly improve thyroid function using multiple linear regression models. The equation can be successfully used by taking the change in FT_4_ as the dependent variable and the use of medication as an independent variable (y = -0.256x + 0.637, F = 3.542). This relationship was also found in the change in TSH levels (y = 0.340x - 0.698, F = 5.047). However, it was difficult to detect changes in blood lipids. After excluding the prepregnancy BMI, a model for TC was established (y = 0.187x + 0.438, F = 3.054).

## Discussion

The present study focused more on the alterations of lipid profiles in patients with hypothyroidism than previous studies. We found that the degree of abnormal lipid metabolism in patients with IH patients was more pronounced, mainly in the form of higher levels of TC, TG, LDL-C, and Apo B. Previous research showed that FT_4_ and TSH play separate functions in lipid metabolism [28, 29]. IH is characterized by normal range TSH levels and only a low level of FT_4_. In the control group, we observed a negative association between FT_4_ and TG levels, which was more significant in patients with IH. Our findings are consistent with those of another cross-sectional study that found a negative association between TG and normal range FT_4_ levels in 2315 euthyroid adults [30]. However, some studies have noted that variations in TC are associated with variations in TSH, regardless of whether FT_4_ levels were normal or abnormal [31]. However, this phenomenon was not observed based on the present study. This may be related to the fact that IH is associated with a slight degree of hypothyroidism and a small sample size.

Experts have begun to explore the relationship between BMI and thyroid function. Evidence suggests that changes in thyroid hormone levels result from weight change rather than a cause [4]. A negative relationship between normal-range FT_4_ levels and BMI was found in a large-sample retrospective study [1]. A study in China reported a negative correlation between FT_4_ levels and prepregnancy BMI in patients with IH [32]. The close relationship between hypothyroidism and dyslipidemia with obesity indicates that the association between maternal thyroid function and metabolic markers may also be mediated by obesity [1]. However, there is no clear evidence supporting this inference. In our study, pregnant women with IH had a higher prepregnancy BMI than controls. Although no specific link was identified, stratification based on BMI levels revealed that pregnant women with a higher prepregnancy BMI (those who were relatively overweight) had lower FT_4_ levels. Due to the nonavailability of all individuals' prepregnancy thyroid function results, we could only hypothesize that those with a higher prepregnancy BMI had a higher risk of developing IH during pregnancy, consistent with previous research. This is partly because obesity increases the levels of adipokines expressed by adipocytes, such as leptin and adiponectin. Leptin can affect the hypothalamic-pituitary-thyroid axis via the JAK2/STAT3 pathway, influencing thyroxine secretion [33, 34]. ΔBMI and GWG reflect, to some extent, maternal fluid expansion, fat accumulation, and placental and fetal growth, and appropriate gain is necessary [34]. We found that patients with IH had a higher ΔBMI and GWG, which was associated with a lower maternal basal metabolism rate. These findings suggest that attention should be given to healthy dietary habits, which reduce the risk of developing thyroid dysfunction during pregnancy. Additionally, weight control should be encouraged throughout pregnancy.

After excluding the disturbance of prepregnancy BMI, we found that in patients with IH, a linear relationship between FT_4_ and TG still existed and was more significant, further confirming that low FT_4_ levels may interfere with lipid metabolism alone in pregnant women. Simultaneously, the difference in ΔBMI and GWG remained, indicating that pregnancy weight gain was relatively high in patients with IH, however, it was unrelated to the initial BMI. Pop also found that pregnant women may experience low FT_4_ levels when they gain weight too quickly, even with a normal prepregnancy BMI [34]. These studies suggest that weight management becomes more complicated during pregnancy in women with low FT_4_ levels. Perhaps thyroid function should also be considered in those who have difficulty managing their weight during pregnancy or have abnormally high lipid indicators.

Age [31] was positively correlated with TSH [35] and negatively correlated with FT_4_ in individuals with normal thyroid function; however, serum lipid levels tended to increase with age. Therefore, age is an independent factor affecting thyroid function and lipid metabolism. However, in our study, the occurrence of IH was independent of age, probably because the participants were fertile women, and the age was relatively concentrated. There is no clear evidence that gravidity and parity affect the occurrence of IH during pregnancy. Therefore, more factors still need to be explored in further prospective, large-sample, multicenter studies.

We established that despite the absence of medication in the dietary treatment group, the data proved that getting more iodine-containing food, such as kelp, could slightly improve thyroid function, mainly by increasing FT_4_ levels. This phenomenon may indicate that, in IH, the alteration of FT_4_ levels after small amounts of iodine supplementation may be more sensitive than that of TSH. After L-T_4_ intervention, the levels of FT_4_ and TSH greatly improved and were more significant than those of the other groups. Although TSH levels were normal, the L-T_4_ treatment reduced TSH levels. These results confirm the effectiveness of L-T_4_ in the treatment of thyroid function.

We investigated the effects of L-T_4_ treatment on the blood lipid profile and the possible link between thyroid function recovery and lipid metabolic changes. This is because traditional lipid-lowering drugs are not the preferred treatment for hyperlipidemia during pregnancy [27]. Based on the treatment group’s lowest raised TC levels, L-T_4_ intervention can partly hinder the elevation of physiological cholesterol in pregnancy. This finding demonstrates that serum TC levels during pregnancy are sensitive to thyroid regulation. Furthermore, we found that both interventions marginally reduced LDL-C by boosting thyroid function, however, L-T_4_ did not show a distinct therapeutic impact. Changes in TG levels in the IH group were greater than those in controls. This could be because of the poor overall thyroid function on retesting in late pregnancy compared with controls, despite intervention with L-T_4_ or dietary iodine supplementation. We hypothesized that the long-term effects of decreased FT_4_ levels on lipids would outweighe the benefits of better thyroid function in terms of lipid alleviation. L-T_4_ has been shown to directly activate the expression of the LDL receptor and cholesterol 7 alpha-hydroxylase (CYP7A1) and stimulate the breakdown of LDL-C and cholesterol into bile acids in the liver, returning partial lipid levels to normal levels [26, 36]. This may be due to the direct effects of TH and TSH on TC and LDL-C regulation [37, 38] and their indirect effects on TG [39–41]. We found that after thyroid function improved, TC and LDL-C levels returned to normal first, while TG lagged.

We further explored the factors influencing the therapeutic effects of L-T_4_. First, stratification based on FT_4_ and TSH levels indicated that the baseline level of thyroid function could partly affect the therapeutic effect of L-T_4_. This means that the more severe the low FT_4_ and high TSH levels are, the greater the improvement in thyroid function following L-T_4_ intervention, as seen in patients with clinical hypothyroidism (CH) and subclinical hypothyroidism (SCH) [42, 43]. Currently, there is no diagnostic standard for hyperlipidemia during pregnancy [44]. However, we used the 50^th^ percentile of lipid distribution to establish subgroups to determine the relative lipid levels. In the subgroup with high TG levels (TG ≥ 2.41 mmol/L), the treatment effect was relatively poor, with less improvement in thyroid function and a higher degree of dyslipidemia. Despite not finding statistically significant differences and the lack of consensus on the lipid-lowering effect of L-T_4_ in patients with IH, we believe there is a connection between the therapeutic effect and the severity of the disease, with the main factors being FT_4_, TSH, and TG levels before treatment.

The present study has several strengths. These results were adjusted for known or potential confounders to minimize errors. And it was devoted to an in-depth exploration of lipid alterations in patients with IH during pregnancy. At the same time, this study was the first study to explore the effect of L-T_4_ on these patients in this region, proved that drug treatment has a certain degree of improvement in some metabolic indicators to a certain extent, which was of great significance for guiding the monitoring and treatment of IH in clinical work. However, as this was a single-center study, caution should be exercised when extrapolating the results to the general pregnant population. This study had some other limitations in data collection: the cases included in this study may have had both thyroid function and blood lipid profile results in the second and third trimesters, affecting the accuracy of calculation for the incidence and consequently impacting the experimental results. Moreover, maternal weight was not included as a metabolic indicator, making it difficult to further speculate on the role of maternal weight factors in the alteration of thyroid function and serum lipids. In the future, this center will conduct prospective cohort studies and test some adipokines to further explore the mechanism. We only studied the effects of levothyroxine treatment on lipid metabolism in IH during pregnancy. However, whether all patients will benefit from levothyroxine therapy, including improving pregnancy outcomes and offspring neurointellectual development, determining the need for clinical intervention and long-term prognosis, or whether such therapy should be given to specific subgroups, remains a major question, and no definitive recommendation can be made at this time [2]. The potential side effects of medicine should also be considered, which were not identified in this study.

## Conclusion

In conclusion, routine thyroid function screening should be carried out for all pregnant women, especially those with high prepregnancy BMI and who have difficulty in weight management or hyperlipidemia during pregnancy. In addition to the physiological elevation of blood lipids during pregnancy, IH pregnant women would show more obvious dyslipidemia. We did observe that levothyroxine had a certain improvement effect on thyroid function and serum lipid metabolism in these patients. Further prospective randomized controlled studies with large samples are needed to determine whether interventions can improve pregnancy outcomes and neurocognitive outcomes in their offspring.

## Data Availability

The datasets analyzed during the current study are not publicly available because they are also part of an ongoing study but are available from the corresponding author on reasonable request.

## Abbreviations

FT_4_: Free thyroxine
IH: Isolated hypothyroidism
L-T_4_: Levothyroxine treatment
TSH: Thyroid stimulating hormone
TG: Triglyceride
TC: Total cholesterol
HDL-C: High-density lipoprotein cholesterol
LDL-C: Low-density lipoprotein cholesterol
APO A1: Apolipoprotein A1
APO B: Apolipoprotein B
ΔBMI: Gestational BMI gain
GWG: gestational weight gain
ATA: American Thyroid Association
HT: Hyothyroxinemia
GDM: gestational diabetes mellitus
ICP: intrahepatic cholestasis of pregnancy
TgAb: thyroglobulin antibody
TPOAb: thyroid peroxidase antibody
T_3_: triiodothyronine
T_4_: tetraiodothyronine
CYP7A1: Cholesterol 7 Alpha Hydroxylase
